# Planar Optode Imaging Reveals Spatio-Temporal Heterogeneity of Rhizosphere Microecology in *Celosia argentea* Under Cadmium Stress

**DOI:** 10.3390/toxics14010034

**Published:** 2025-12-27

**Authors:** Yunpeng Ge, Kaiyang Ying, Songhao Zhang, Shenglei Wang, Yayu Fang, Jing Huang, Hua Lin, Ting Xu, Guo Yu

**Affiliations:** 1 Guangxi Key Laboratory of Calcium Carbonate Resources Comprehensive Utilization, College of Materials and Chemical Engineering, Hezhou University, Hezhou 542899, China; 2 Guangxi Key Laboratory of Environmental Pollution Control Theory and Technology, Guilin University of Technology, Guilin 541006, Chinalinhua5894@163.com (H.L.); 3 Zhejiang Engineering Survey and Design Institute Group Co., Ltd., Ningbo 315012, China; 4 Guangxi Qichuan Spatial Information Technology Co., Ltd., Nanning 530200, China; 18878996083@163.com; 5 Guangxi Kangwanjia Farmland Soil Remediation Co., Ltd., Nanning 530601, China; 6 Center for Water and Ecology, State Key Laboratory of Iron and Steel Industry Environmental Protection, School of Environment, Tsinghua University, Beijing 100084, China; 7 University Engineering Research Center of Watershed Protection and Green Development, Guangxi, Guilin University of Technology, Guilin 541006, China; 8 Key Laboratory of Carbon Emission and Pollutant Collaborative Control, Education Department of Guangxi Zhuang Autonomous Region, Guilin University of Technology, Guilin 541006, China

**Keywords:** phytoremediation, rhizosphere, heavy metal, planar optode, hyperaccumulator

## Abstract

Understanding rhizosphere microscale processes is essential for evaluating plant–soil interactions under heavy metal stress. In this study, planar optode imaging was used to investigate the spatio-temporal distribution of O_2_, pH, and CO_2_ in the rhizosphere of *Celosia argentea*, a Cd hyperaccumulator, grown in Cd-contaminated and uncontaminated soils. The results demonstrated pronounced spatial heterogeneity, with O_2_ hotspots concentrated near root surfaces, localized rhizospheric alkalinization at root tips, and elevated CO_2_ levels reflecting active root metabolism. Under Cd stress, O_2_ levels were initially suppressed, while pH and CO_2_ increased, indicating adaptive physiological responses. As plant growth progressed, O_2_-enriched zones expanded, pH elevation persisted, and CO_2_ efflux continued, suggesting coordinated regulation of the rhizospheric microenvironment. These changes may influence microbial activity and nutrient dynamics in the rhizosphere, potentially supporting root function and plant adaptation under metal stress. This study provides mechanistic insights into root-induced microenvironmental regulation under Cd stress and demonstrates the potential of planar optode imaging for assessing plant-driven remediation processes in contaminated soils.

## 1. Introduction

Heavy metal contamination, particularly cadmium (Cd), represents a severe environmental hazard due to its high toxicity, mobility, and persistence in soil systems [1,2]. Cd accumulation in agricultural soils not only impairs soil ecological functions but also poses substantial risks to food security and human health [3,4]. Understanding how plants and their associated rhizosphere environments respond to Cd stress is therefore critical for developing effective remediation strategies.

The rhizosphere is a dynamic microenvironment characterized by steep gradients of physicochemical parameters such as oxygen (O_2_), carbon dioxide (CO_2_), and pH, which strongly influence microbial activity, nutrient cycling, and metal mobility [5]. Spatio-temporal heterogeneity in these parameters determines root–soil interactions and plays a pivotal role in regulating metal bioavailability [6,7,8]. However, conventional bulk soil measurements often obscure this fine-scale variability, limiting mechanistic understanding of plant–soil–metal interactions under Cd stress [9,10].

Planar optodes provide a powerful imaging tool to capture the two-dimensional distribution and temporal dynamics of key rhizosphere parameters at high spatial resolution [11,12]. By visualizing gradients of O_2_, CO_2_, and pH in situ, planar optodes enable the direct observation of how root activity and metal stress reshape the microecological environment at the root–soil interface [13,14]. Despite their advantages, applications of planar optodes to investigate rhizosphere processes under heavy metal stress remain limited, especially in naturally contaminated soils.

Hyperaccumulator plants often adapt to metal stress by actively modifying rhizosphere conditions, including the secretion of organic acids, modulation of pH, and localized oxygen release, which collectively facilitate metal detoxification, nutrient acquisition, and microbial interactions [14,15]. *Celosia argentea*, a Cd hyperaccumulator with strong remediation potential, offers a valuable model for elucidating rhizosphere responses to Cd exposure [16,17]. This study represents one of the first attempts to visualize rhizosphere O_2_, pH, and CO_2_ dynamics in Cd-contaminated soils using planar optode imaging.

In this study, planar optodes were employed to visualize the dynamic distribution of O_2_, CO_2_, and pH in the rhizosphere of *C. argentea* under Cd stress. The objectives were to (i) characterize the spatio-temporal variations of rhizosphere microecological parameters, (ii) reveal how Cd stress modifies these gradients, and (iii) advance the understanding of root–soil interactions that regulate Cd bioavailability. The findings provide important mechanistic insights into rhizosphere processes under heavy metal stress and contribute to the development of plant-based strategies for the remediation of Cd-contaminated soils.

## 2. Material and Methods

### 2.1. Plant Cultivation and Soil Preparation

Seeds of *C. argentea* were collected from Sidi Village, Xingping Town, Yangshuo County, Guilin, China. Seeds were surface-sterilized with 2% NaClO for 5 min, rinsed thoroughly with deionized water, and sown in seedling trays filled with sterilized quartz sand. Trays were maintained in a greenhouse at 20–30 °C and ~60% relative humidity under a natural light regime with supplemental illumination to ensure a 14 h photoperiod. Seedlings emerged within 3 days and were watered daily with deionized water. From the second week onward, they were irrigated once per week with 50% Hoagland’s nutrient solution. When plants developed 4–6 true leaves and reached 5–6 cm in height, uniform seedlings were transplanted into rhizoboxes for further experiments.

Two types of soils were used: (i) uncontaminated soil collected from the Yanshan Campus of Guilin University of Technology (25°03′41.05″ N, 110°18′17.15″ E), and (ii) Cd-contaminated agricultural soil collected near farmland in Sidi Village, Xingping Town, Yangshuo County, Guilin (24°40′35.09″ N, 110°31′39.20″ E). The soil was collected from the 0–20 cm surface layer. Sidi Village is located adjacent to a lead–zinc tailing area, where the agricultural soils have been contaminated by Cd due to long-term mining activities [17]. Soils were air-dried at room temperature, gently disaggregated, and passed through a 2 mm sieve to remove stones, coarse aggregates, and plant residues. Subsamples were analyzed for physicochemical properties, including pH, organic matter content, particle size distribution, cation exchange capacity, and total as well as available metal concentrations, following standard analytical protocols recommended by the Soil Science Society of China and the Chinese Society of Agrochemistry [18]. The pH was measured with a pH meter in a 1:2.5 (soil/water) aqueous suspension after shaking for 1 h. The cation exchange capacity was measured using a spectrophotometer after extraction with hexaammonium cobalt trichloride. The total nitrogen content was determined by the Kjeldahl method. The total Cd concentration was measured by inductively coupled plasma optical emission spectrometry. Selected physicochemical properties of the soils are listed in Table 1.

### 2.2. Rhizobox Experiment Design

Custom-made acrylic rhizoboxes (Zhongke Zhigan Environmental Technology Co., Nanjing, China) were used to investigate the rhizosphere microecology of *C. argentea*, following established designs for in situ root–soil interface studies [19,20]. Each rhizobox measured 10 cm × 8 cm × 20 cm (length × width × height) internally, with one detachable side secured by plastic screws to avoid potential metal contamination and a 3 mm-thick plastic gasket inserted between the panel and main frame to ensure an airtight seal [21]. Two soil treatments were established—uncontaminated soil (Control) and Cd-contaminated soil (Cd) as described in Section 2.1—with 1.5 kg of the corresponding soil added to each rhizobox, lightly moistened with deionized water, homogenized, and gently compacted to achieve uniform density. Rhizoboxes were labeled according to treatment and the parameter to be monitored (DO, pH, or CO_2_). For each treatment, three independent rhizoboxes were prepared as biological replicates. One uniform *C. argentea* seedling of similar height and leaf number was selected and transplanted into the center of each rhizobox to minimize individual variation. The number of biological replicates was determined based on previous rhizobox-based studies. Soil moisture was maintained at 70–75% of the soil’s water-holding capacity throughout the experiment by regular weighing and watering, and the rhizoboxes were positioned at a 45° angle in a greenhouse under controlled conditions (25 °C daytime/20 °C night; 70–75% relative humidity; 14 h photoperiod; light intensity 300–400 μmol photons m^−2^ s^−1^), with the exterior wrapped in aluminum foil to simulate dark rhizosphere conditions and prevent light interference [22]. No additional fertilizers were applied during the imaging period to avoid altering rhizosphere chemistry. After an initial establishment period, rhizoboxes with the most uniform and vigorous seedlings were chosen for planar optode imaging.

### 2.3. Planar Optode Imaging of Rhizosphere Parameters

High-resolution planar optodes (EasySensor, China) were employed to monitor rhizosphere O_2_, pH, and CO_2_ dynamics in *C. argentea*, as described in our previous study [6,23]. A xenon lamp with adjustable wavelength served as the excitation light source, with wavelengths set to 425 nm for pH, 415 nm for dissolved oxygen (DO), and 470 nm for CO_2_, and corresponding bandpass filters placed in front of the camera lens to selectively capture the desired fluorescence signals [24]. To minimize interference from reflections on the rhizobox walls, a black light-blocking shield was positioned 2 cm behind the sensor foil. Fluorescence images were acquired using a high-resolution digital camera mounted on the planar optode system, with imaging performed daily at 14:00 at 24 h intervals over a continuous period of 10 days. All measurements were conducted in a darkroom to prevent ambient light interference, ensuring consistent conditions. Although O_2_, pH, and trace metal measurements were conducted on separate occasions, potential effects of root growth between experiments were minimized by shortening the intervals between measurements. During removal of the detachable rhizotron windows, the rhizotron was carefully laid flat, and the upper section containing the plant was sealed with plastic film to prevent water loss, ensuring minimal disturbance to the rhizosphere microenvironment. Three biological replicates per treatment were imaged to account for plant-to-plant variability, and all measurements were performed under identical environmental conditions to minimize external variation. The acquired images were processed using the Fiji version of ImageJ (National Institutes of Health, USA), and spatial distribution maps were generated based on calibration curves: DO was mapped between 0% and 100% saturation, pH between 6.0 and 9.0 with at least six calibration points, and CO_2_ between 0 and 40 matm with at least eight calibration points. Iso-concentration contour maps were subsequently produced to visualize the spatio-temporal heterogeneity of each rhizosphere parameter.

### 2.4. Data Processing and Statistical Analysis

Fluorescence images acquired from the planar optode system were first processed using ImageJ to correct for background signals and standardize fluorescence intensity across all time points [25]. Regions of interest (ROIs) corresponding to the rhizosphere were manually delineated for each rhizobox based on root position and sensor coverage. Within each ROI, average values of dissolved oxygen, pH, and CO_2_ were calculated, and pixel-by-pixel intensity data were used to assess local heterogeneity. Temporal dynamics were evaluated by comparing measurements across the 14-day imaging period, while spatial variations were visualized through iso-concentration contour maps generated in ImageJ, highlighting gradients and micro-scale heterogeneity of rhizosphere parameters.

For quantitative analysis, statistical metrics including mean, standard deviation, coefficient of variation, and range were computed using OriginPro 2022 (OriginLab, Northampton, MA, USA) for each parameter and treatment. Differences between treatments (Control vs. Cd) and among replicate rhizoboxes were assessed using one-way ANOVA followed by Tukey’s post hoc test, with significance set at *p* < 0.05. Additionally, temporal trends were analyzed to identify peak or minimum values of O_2_, pH, and CO_2_, and correlation analyses were conducted to explore interdependencies among rhizosphere parameters over time.

## 3. Results

### 3.1. Spatio-Temporal Dynamics of O_2_ in the Rhizosphere

**Figure 1:**
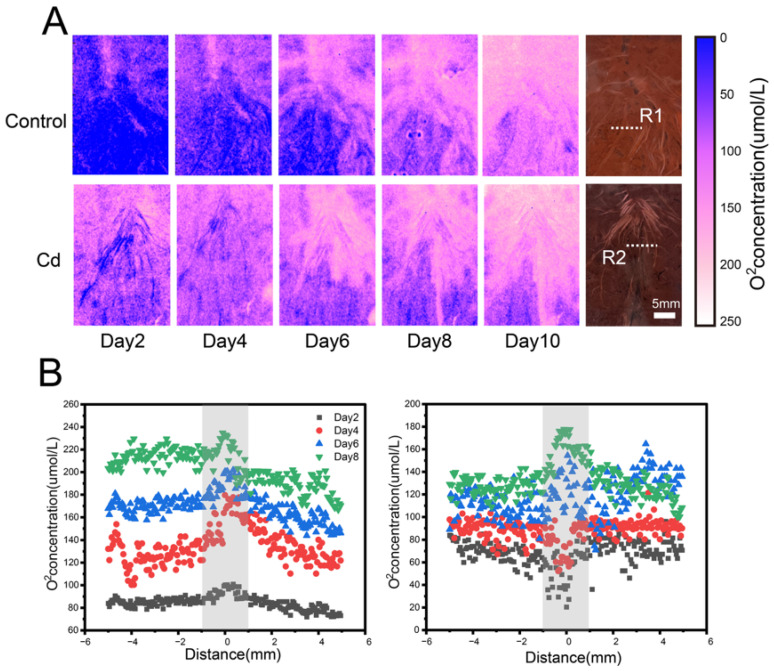
In situ high-resolution imaging of the rhizospheric O_2_ dynamics of *C. argentea* using planar optode. (**A**) Two-dimensional spatial distribution of O_2_ in the control and Cd-treated groups. (**B**) Scatter plot illustrating O_2_ spatial patterns in the rhizosphere of *C. argentea* in the control (**on the left**) and Cd-treated groups (**on the right**). Gray shading indicates the rhizosphere zone. R1 and R2 indicate the selected regions of interest used for quantitative analysis.

The spatial distribution of O_2_ in the rhizosphere of *C. argentea* was highly heterogeneous, exhibiting pronounced spatial variability across treatments (Figure 1). In the control group, O_2_ hotspots were predominantly localized around the root surfaces, whereas the root tips consistently exhibited lower O_2_ levels than the non-apical regions. In contrast, the Cd treatment displayed a distinct temporal pattern. During days 2–4, the rhizospheric O_2_ concentrations of Cd-exposed plants were markedly lower than those in the non-rhizosphere soil. As plant growth progressed, the area of O_2_ hotspots gradually expanded, and by day 6, the O_2_ concentration around roots in the Cd-contaminated soil surpassed that of the surrounding soil, exhibiting a pattern similar to the control group. Quantitatively, the rhizospheric O_2_ concentrations in the control group ranged from 85.6 to 232.3 μmol·L^−1^, while those in the Cd treatment ranged from 20.4 to 175.6 μmol·L^−1^. Overall, rhizospheric O_2_ concentrations were substantially higher than those in the bulk soil, with O_2_ levels declining sharply with increasing distance from the root surface. In the control group, O_2_ concentrations at the root center were 1.16–1.47 times greater than those measured 5 mm away (*p* < 0.05). In contrast, in the Cd-contaminated soil, O_2_ levels were initially lower in the root region but became 1.28–1.45 times higher than those in non-rhizosphere zones after day 6. For example, on day 6, the O_2_ concentration at the root center (200.47 μmol·L^−1^) was 1.16-fold higher than that 5 mm from the root surface (173.1 μmol·L^−1^).

### 3.2. Spatio-Temporal Dynamics of pH in the Rhizosphere

**Figure 2:**
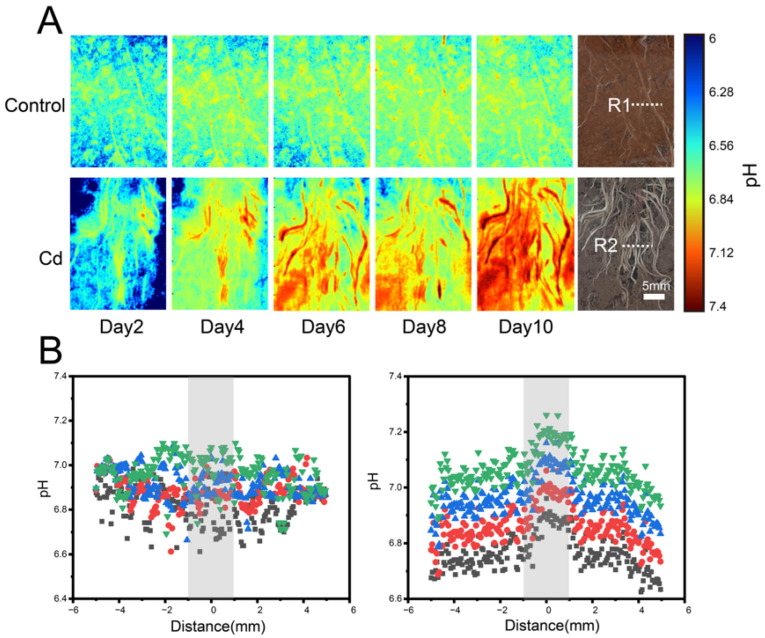
In situ high-resolution imaging of rhizospheric pH dynamics of *C. argentea* using planar optode. (**A**) Two-dimensional spatial distribution of pH in the control and Cd-treated groups. (**B**) Scatter plot illustrating pH spatial patterns in the rhizosphere of *C. argentea* in the control (**on the left**) and Cd-treated groups (**on the right**). Gray shading indicates the rhizosphere zone. R1 and R2 indicate the selected regions of interest used for quantitative analysis.

The spatial distribution of rhizospheric pH in *C. argentea* was highly heterogeneous, showing clear variation across different soils (Figure 2). Overall, the pH values increased in the vicinity of the roots. In the control group, the change was modest, with rhizospheric pH ranging from 6.8 to 7.02 throughout the experimental period. In contrast, Cd-exposed plants exhibited more pronounced fluctuations, with rhizospheric pH ranging from 6.69 to 7.34. Significant pH elevation was observed at the root tips during plant growth (*p* < 0.05), indicating localized alkalinization in the surrounding soil. Over time, rhizospheric pH showed a consistent upward trend in all soils. Specifically, in the control group, root center pH increased from 6.86 on day 2 to 7.02 on day 8, an increase of 0.16 units. In the Cd treatment, pH rose from 6.95 to 7.34, a total increase of 0.39 units.

### 3.3. Spatio-Temporal Dynamics of CO_2_ in the Rhizosphere

**Figure 3:**
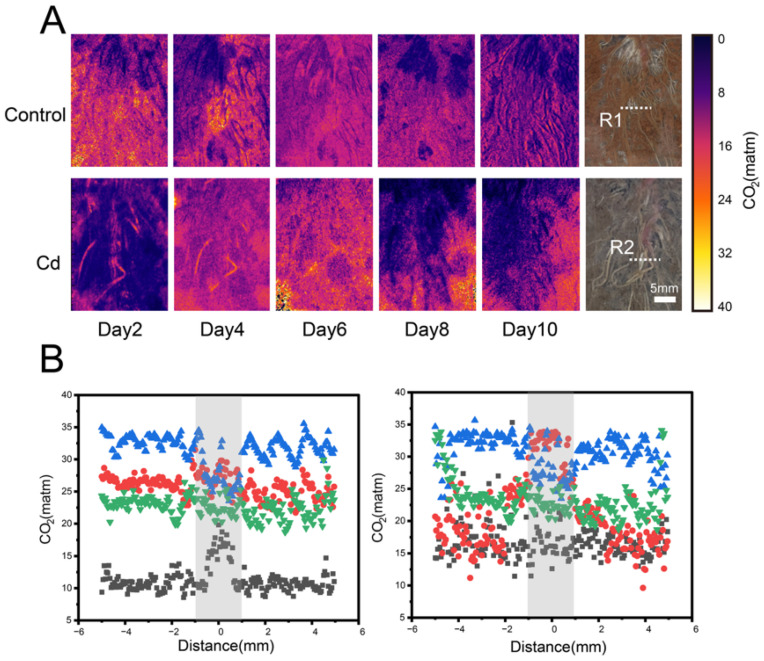
In situ high-resolution imaging of rhizospheric CO_2_ dynamics of *C. argentea* using planar optode. (**A**) Two-dimensional spatial distribution of CO_2_ in the control and Cd-treated groups. (**B**) Scatter plot illustrating CO_2_ spatial patterns in the rhizosphere of *C. argentea* in the control (**on the left)** and Cd-treated groups (**on the right**). Gray shading indicates the rhizosphere zone. R1 and R2 indicate the selected regions of interest used for quantitative analysis.

The spatial distribution of rhizospheric CO_2_ in *C. argentea* was highly heterogeneous (Figure 3). During the experimental period, CO_2_ hotspots in the control group were primarily concentrated around the root zone, particularly during days 2–4. At this stage, CO_2_ concentrations at the root tips were higher than in the non-apical regions. The Cd treatment showed a similar trend, with root-associated CO_2_ consistently exceeding that of the surrounding soil. By day 6, CO_2_ concentrations in the control group began to decline gradually, whereas in the Cd-contaminated soil, a noticeable decrease was observed around day 8. Throughout the experiment, rhizospheric CO_2_ levels ranged from 6 to 35 matm in both treatments. Overall, rhizospheric CO_2_ concentrations were substantially higher than those in bulk soil, and declined with increasing distance from the root tips. In the control group, the maximum CO_2_ concentration at the root center reached 29.12 matm, whereas in the Cd-contaminated soil, it reached 33.95 matm. Between days 2 and 10, CO_2_ concentrations first increased and then decreased in both treatments. For instance, in the control group, root center CO_2_ levels were 19.82 matm on day 2, peaked at 29.12 matm on day 4, and then declined to 22.4 matm on day 6 and 21.85 matm on day 8. The Cd-contaminated soil exhibited a similar pattern.

## 4. Discussion

As a Cd hyperaccumulator, *C. argentea* actively modulates its rhizospheric microenvironment in response to Cd stress, thereby influencing processes that govern Cd mobility and stabilization in soils [26,27]. In soil systems, the concentrations of O_2_ and CO_2_ are pri-marily determined by the balance between biological production or consumption (root and microbial respiration), physical diffusion through soil pores, and soil structural properties such as porosity and water content [28]. The spatio-temporal dynamics of O_2_, pH, and CO_2_ reveal that these parameters are closely interrelated and collectively shape soil physicochemical conditions under Cd stress [29]. Rhizospheric O_2_ exhibited pronounced spatial heterogeneity, with hotspots concentrated near root surfaces, reflecting the root radial oxygen loss [30]. During early Cd exposure, O_2_ levels were transiently suppressed, likely due to Cd-induced inhibition of root respiration and membrane integrity. As plants grew, adaptive responses—including enhanced aerenchyma formation and upregulated antioxidant activity—restored root metabolic function, increased ROL, and expanded oxygenated microzones [31,32]. Early suppression of O_2_ under Cd stress is consistent with reports that Cd impairs root respiration and membrane integrity [33], while the subsequent recovery may reflect adaptive increases in root porosity or metabolic reprogramming that restore aerobic capacity [34]. Notably, localized oxygenation can alter redox-sensitive reactions in the rhizosphere, potentially promoting the oxidation of reduced Fe and Mn phases [35,36]. However, the magnitude and spatial extent of these processes are also likely influenced by background soil properties. In Cd-contaminated soils with reduced organic matter content, microbial respiration rates and associated O_2_ consumption may be lower, whereas soils enriched in organic substrates may exhibit enhanced microbial activity and stronger O_2_ depletion [37]. Although Fe/Mn plaque formation and Cd speciation were not directly measured in this study, such redox-driven processes are widely recognized as important regulators of trace metal partitioning and may vary depending on soil nutrient and organic matter status.

Concurrently, rhizospheric pH increased, particularly at the root tips, reflecting active secretion of alkaline compounds to buffer Cd-induced acidity and maintain metabolic homeostasis [38]. Mechanisms driving pH increases may include reduced H^+^ efflux, selective cation uptake, or secretion of alkaline exudates [39]. Elevated pH synergistically interacted with O_2_-enriched microzones to favor Cd precipitation and complexation, further stabilizing metal speciation [6,40]. CO_2_ efflux from active root respiration mirrored root metabolic activity, with elevated levels near root tips supporting nutrient cycling and stimulating microbial activity [41]. CO_2_ hotspots can feed back to local carbonate chemistry and influence pH microgradients, while also serving as a labile carbon source that reshapes microbial community structure and enzyme activities [42,43]. Notably, rhizospheric microbial communities are known to be highly sensitive to CO_2_ and pH gradients. Based on previous studies, oxygenated and alkalinized microzones may favor the abundance and activity of aerobic and pH-tolerant microorganisms, which could potentially contribute to enhanced nutrient turnover, metal immobilization, and the maintenance of root–soil homeostasis [44,45]. During early Cd exposure, the observed suppression of O_2_ together with elevated CO_2_ may indicate a tight coupling between root respiration and microbial metabolism in the rhizosphere, while pH modulation might play a buffering role in alleviating metal toxicity [46]. Although this interpretation is consistent with established concepts of root–microbe interactions under heavy metal stress, further studies incorporating direct measurements of root metabolic activity and microbial community dynamics are required to confirm these mechanisms.

Collectively, these findings demonstrate that *C. argentea* actively regulates its rhizospheric environment through the coordinated regulation of O_2_, pH, and CO_2_, creating favorable conditions for both root function and microbial activity. The interplay among these parameters reduces Cd mobility and bioavailability, sustains root metabolic homeostasis, and potentially enhances phytoremediation efficiency. This dynamic, integrated control of the rhizosphere underscores the critical role of plant–microbe–soil interactions in stabilizing heavy metals and provides mechanistic insights for optimizing plant-based strategies for contaminated soil remediation.

## 5. Conclusions

*C. argentea* actively modulates its rhizospheric microenvironment through coordinated regulation of O_2_, pH, and CO_2_, creating oxygenated, alkalinized, and metabolically active zones that support root physiological activity and stimulate microbial processes. Under Cd stress, adaptive changes—including initial suppression and subsequent recovery of O_2_, sustained pH elevation, and active CO_2_ efflux—demonstrate the plant’s capacity to maintain rhizospheric homeostasis. These coordinated microenvironmental adjustments reduce Cd mobility and bioavailability, sustain root metabolic function, and enhance phytoremediation efficiency. This study demonstrates the potential of planar optode imaging as a powerful tool to visualize and quantify root-induced microenvironmental dynamics governing heavy metal behavior in soils. Overall, the findings highlight the critical role of hyperaccumulator-driven rhizosphere regulation in supporting plant growth, microbial activity, and the effective remediation of Cd-contaminated soils, providing mechanistic insights for optimizing plant-based remediation strategies. Future research integrating planar optode imaging with microsensor-based profiling and synchrotron spectroscopic analyses could further elucidate the coupled dynamics of rhizosphere redox processes and Cd speciation.

## Figures and Tables

**Table 1:** Physicochemical properties of the two soil samples.

Index	Non-Contaminated Soil	Cd Contaminated Soil
pH	6.60 ± 0.15	6.85 ± 0.08
Organic matter (g/kg)	4.13 ± 0.15	8.33 ± 0.40
Cation exchange capacity (cmol kg^−1^)	4.50 ± 0.06	10.36 ± 0.20
Total N (g/kg)	1.36 ± 0.02	4.30 ± 0.03
Total Cd (mg/kg)	0.36 ± 0.03	4.03 ± 0.16
Textural class	Sandy clay loam	Sandy clay loam

## Data Availability

The original contributions presented in this study are included in the article. Further inquiries can be directed to the corresponding authors.
